# West Nile Virus Transmission in Resident Birds, Dominican Republic

**DOI:** 10.3201/eid0910.030222

**Published:** 2003-10

**Authors:** Oliver Komar, Mark B. Robbins, Kaci Klenk, Bradley J. Blitvich, Nicole L. Marlenee, Kristen L. Burkhalter, Duane J. Gubler, Guillermo Gonzálvez, Carlos J. Peña, A. Townsend Peterson, Nicholas Komar

**Affiliations:** * University of Kansas, Lawrence, Kansas, USA; † Centers for Disease Control and Prevention, Fort Collins, Colorado, USA; ‡ Colorado State University, Fort Collins, Colorado, USA; § Centro Nacional de Control de Enfermedades Tropicales, Santo Domingo, Dominican Republic

## Abstract

We report West Nile virus (WNV) activity in the Dominican Republic for the first time. Specific anti-WNV antibodies were detected in 5 (15%) of 33 resident birds sampled at one location in November 2002. One seropositive bird was <4 months old, indicating a recent infection.

The initial outbreak of West Nile virus (WNV; family *Flaviviridae,* genus *Flavivirus*) in the Western Hemisphere took place in New York in 1999, with deaths observed in humans, horses, and numerous species of wild birds (*1*). Since then, this virus has spread rapidly across North America (*2*,*3*). Migratory birds are suspected of being responsible for the rapid spread of WNV through North America (*4*), and transport of WNV by Neotropical migratory birds throughout the New World has been anticipated (*5*).

Although WNV has spread rapidly through continental areas, its ability to spread across oceanic barriers is uncertain. The many islands of the West Indies represent the wintering grounds of numerous North American migratory birds (*6*,*7*) that breed in or migrate through WNV transmission foci in the United States. The Caribbean islands tend to have high human population density, and low populations of many birds and other vertebrates are restricted to certain islands. Introduction of WNV to the West Indies would present a human and equine health concern and potentially threaten numerous endangered and endemic bird species and perhaps other wild vertebrates.

Given the speculation that WNV may be disseminated by migrating birds (*5*,*8*,*9*), we hypothesized that the virus would be introduced to the Dominican Republic. Accordingly, we sampled apparently healthy birds there for evidence of locally acquired WNV infection.

## The Study

Birds were studied at two sites in the Dominican Republic, on the island of Hispaniola (Figure): Parque Nacional Sierra de Baoruco (November 7–16, 2002; 18° 12' N, 71° 32' W) and Parque Nacional Los Haitises (November 18–23, 2002; 19° 00' N, 69° 30' W). Birds were collected by standard methods (*10*). Tissues (eye, spleen, and kidney) were removed from 89 birds of 29 species (25 resident, 4 migratory) at Sierra de Baoruco and from 58 birds of 27 species (18 resident, 9 migratory) at Los Haitises; the tissues were tested for active WNV infection. Blood samples were collected from a subsample of these birds, including 41 that represented 18 resident species at Sierra de Baoruco and 33 that represented 16 resident species at Los Haitises. Blood was not collected from migratory birds or from certain very small resident birds, such as hummingbirds. Blood and tissue specimens were frozen immediately in liquid nitrogen for transportation and then stored at –70°C. Voucher specimens (including additional tissue samples) were prepared for all birds and are deposited at the University of Kansas Natural History Museum (KUNHM).

**Figure:**
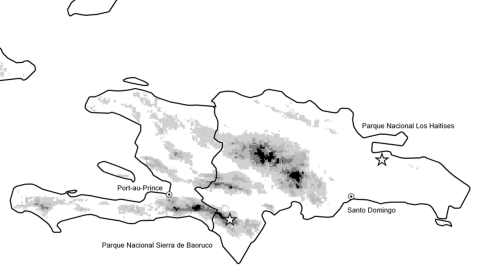
Hispaniola in the West Indies, on which are located Haiti (western third of the island) and the Dominican Republic. West Nile virus transmission occurred at Parque Nacional Los Haitises before November 2002. Shades of gray are 500-m intervals (e.g., 0–500, 500–1000).

The sex and breeding condition of each bird were determined by examination and measurement of gonads. Birds’ ages were assessed by plumage, skull ossification, and presence or absence of a bursa of Fabricius. The migratory or resident status of each bird was determined on the basis of standard references (*6*,*7*). For species that had both migratory and resident populations, we based status assessment on breeding conditions (breeding birds were assumed to be resident).

Serum samples were screened for flavivirus-neutralizing antibodies by plaque-reduction neutralization test (PRNT) according to standard methods (*11*) and by using challenge inocula of 100 plaque-forming units (PFU) WNV strain NY99-4132 and Saint Louis encephalitis virus (SLEV) strain TBH-28. Testing for neutralizing antibody to SLEV was important because this virus has been detected in the Caribbean and isolated from wild birds (*12*) and cross-reacted to anti–WNV-neutralizing antibodies in 6% of seropositive birds sampled in New York (*13*). PRNTs were performed with Vero cells in 6-well plates and a serum dilution of 1:10 in BA1 buffer (Hanks M-199 salts, 0.05 M Tris pH 7.6, 1% bovine serum albumin, 0.35 g/L sodium bicarbonate, 100 U/mL penicillin, 100 mg/L streptomycin, 1 mg/L Fungizone). Specimens that neutralized the virus stocks by at least 80% were further titrated in duplicate. To identify either virus as the causative agent, we used 90% neutralization as the criterion for a positive test result, and a four fold greater titer to one of the flaviviruses was considered diagnostic for that flavivirus.

Serum samples that showed neutralizing antibody titers to WNV or SLEV were tested by epitope-blocking enzyme-linked immunosorbent assays (ELISAs) with the WNV-specific monoclonal antibody 3.1112G, which discriminates between WNV and SLEV infections in birds (*14*). Because of the current lack of information on flaviviruses in the Dominican Republic, we required both the PRNT and the ELISA to test positive to consider a serum sample positive for WNV.

Tissues from each individual bird were pooled and homogenized in 2 mL of BA1 supplemented with 20% fetal bovine serum. Homogenates were clarified by centrifugation at 3,700 rpm for 10 min at 4°C. Four hundred microliters of each homogenate was screened for virus by Vero plaque assay (*11*). Homogenates obtained from flavivirus-seropositive birds were assayed for WNV RNA by TaqMan reverse-transcription polymerase chain reaction with WNV-specific primers (*15*).

Serum samples from nine resident birds tested positive for flavivirus-neutralizing antibodies (Table). Of these, five birds were positive for WNV antibodies by PRNT and blocking ELISA. All WNV antibody–positive birds were sampled at the Los Haitises study site. One serum sample was collected from an immature bird (ruddy quail-dove, *Geotrygon montana*; KUNHM 94667) that was <4 months old, suggesting that virus transmission was recent. Virus was not isolated from any of the tissues tested from 118 resident and 29 migratory birds (all migrants were Parulidae), nor did we detect WNV RNA in any of the tissue homogenates from flavivirus-seropositive birds.

**Table:** Laboratory results for flavivirus-seropositive birds collected in the Dominican Republic, 2002^a^

Species	KUNHM catalog no.	Date sampled	Age and sex^b^	Locality	SLEV PRNT_90_	WNV PRNT_90_	% inhibition by ELISA^c^	Result
Ruddy quail-dove (*Geotrygon montana*)	94667	21 Nov	Immature male	Los Haitises	<10^d^	20^d^	40	WNV
Mangrove cuckoo (*Coccyzus minor*)	94671	19 Nov	Adult female^e^	Los Haitises	<10	160	81	WNV
Hispaniolan lizard cuckoo (*Saurothera longirostris*)	94669	18 Nov	Adult female	Los Haitises	160	640	73	WNV
Hispaniolan lizard cuckoo (*Saurothera longirostris*)	94670	21 Nov	Adult male	Los Haitises	<10	40	23	FLAV
Hispaniolan trogon (*Priotelus roseigaster*)	94951	12 Nov	Adult male	Sierra de Baoruco	<10	10	6	FLAV
Red-legged thrush (*Turdus plumbeus*)	94956	20 Nov	Adult female^e^	Los Haitises	10	160	86	WNV
Red-legged thrush (*Turdus plumbeus*)	94689	19 Nov	Adult male^e^	Los Haitises	<10	80	not available	FLAV
Red-legged thrush (*Turdus plumbeus*)	94691	21 Nov	Adult female	Los Haitises	20	1280	61	WNV
Greater Antillean grackle (*Quiscalus niger*)	94949	19 Nov	Adult male	Los Haitises	640	40	42	FLAV^f^

^a^ELISA, enzyme-linked immunosorbent assay; FLAV, undifferentiated flavivirus; KUNHM, University of Kansas Natural History Museum, Division of Ornithology; PRNT_90,_ reciprocal 90% plaque reduction neutralization titer; SLEV, Saint Louis encephalitis virus; WNV, West Nile virus. ^b^Birds were in nonbreeding condition unless otherwise indicated. ^c^Inhibition values >30% were considered significant. ^d^Values represent reciprocal titers; threshold of detection was 1:10. ^e^Breeding condition, as determined by size of gonads. ^f^Although serologic results based upon PRNT would suggest that this specimen be identified as SLEV antibody–positive, the WNV antibody–positive result in the blocking ELISA indicates that this specimen was possibly positive for both SLEV and WNV. However, secondary flavivirus infections are notorious for heterologous reactivity, so infection by WNV or other flaviviruses causing these reactions could not be ruled out. Hence, the determination as FLAV.

## Conclusions

Our finding of WNV-neutralizing antibodies in five resident birds represents the first evidence of WNV activity in the Dominican Republic. No cases of WNV infection in humans, horses, or birds were known at the time of sampling. The birds in this study could have been infected with WNV in the Dominican Republic as recently as early November 2002; nonetheless, the virus probably arrived earlier in the Caribbean region. Because no current infections were detected, our results reflect past virus transmission activity. Although we cannot determine when this activity began, the seropositive immature quail-dove presumably was infected after mid-July 2002, when it was born, and before early November 2002, in order to have stimulated detectable antibody production by mid-November.

The earliest evidence of WNV transmission in the West Indies is a human case from the Cayman Islands in 2001 (*16*). WNV-seropositive birds captured in Jamaica early in 2002 may have been infected in 2001 or earlier (*17*). Additional evidence of WNV transmission in the Caribbean region includes the report of two seropositive horses in Yucatán state, Mexico, sampled in July 2002 (*18*). Although the seropositivity of vertebrates in Cayman Islands, Jamaica, Mexico, and now the Dominican Republic is strong evidence for WNV activity in the region, it is indirect evidence and does not entirely rule out the possibility of cross-reactions with another flavivirus in laboratory assays. WNV remains to be isolated from the region.

The presence of WNV at Los Haitises may have resulted from transportation by viremic migratory birds from North America, where WNV transmission foci are widespread (*2*). Several migratory bird species, in particular parulid warblers (order Passeriformes), were observed at this site. At least some passerine birds are capable of transmitting virus during their few days of viremia (*19*). Therefore, transmission to mosquitoes or predators from viremic migrants would be possible for a brief period (a few days, at most) after arrival at a site. Virus introduction into Caribbean ecosystems is therefore likely to occur at coastal sites where transoceanic migrants make first landfall.

We found no evidence of active virus in bird tissues of both resident and migratory species. We did not test serum samples from migrants because the presence of antibodies would not be informative, given the history of these birds traveling through areas of WNV transmission in or near North American breeding grounds. We presume that the five seropositive resident birds were infected locally because the four species involved are not migratory (*7*). Although young birds may disperse several kilometers from natal sites (*20*), adults probably live entirely within breeding territories.

Although only five birds (15%, 95% confidence interval [CI] 5% to 32%) at Los Haitises were found to be seropositive, the results suggest that transmission of WNV among bird populations at that site was widespread. For comparison, seroprevalence of WNV-neutralizing antibodies in resident birds was 50% (CI 44% to 57%) in Queens, New York City, after the 1999 outbreak (*13*), and 23% (CI 18% to 29%) in Staten Island after the 2000 outbreak (*21*). We used conservative criteria for determining a positive result because the background diversity of flaviviruses in the Dominican Republic has not been studied recently. If only the PRNT had been used (as was the case in the New York studies), then seven (21%, CI 7% to 35%) of the birds from Los Haitises would have been reported as positive for antibodies to WNV.

The evidence for local WNV transmission in the Dominican Republic indicates risk for West Nile fever and meningoencephalitis in the human, equine, and avian populations of Hispaniola. We suggest that WNV be considered in the differential diagnosis of humans and other vertebrates with central nervous system disease in Hispaniola.
